# Inhibition of p300 by Garcinol Protects against Cisplatin-Induced Acute Kidney Injury through Suppression of Oxidative Stress, Inflammation, and Tubular Cell Death in Mice

**DOI:** 10.3390/antiox9121271

**Published:** 2020-12-14

**Authors:** Jung-Yeon Kim, Jungmin Jo, Jaechan Leem, Kwan-Kyu Park

**Affiliations:** 1 Department of Immunology, School of Medicine, Catholic University of Daegu, Daegu 42472, Korea; jy1118@cu.ac.kr; 2 Division of Hematology-Oncology, Department of Internal Medicine, Ewha Womans University Mokdong Hospital, Seoul 07985, Korea; 10003kj@ewha.ac.kr; 3 Department of Pathology, School of Medicine, Catholic University of Daegu, Daegu 42472, Korea; kkpark@cu.ac.kr

**Keywords:** cisplatin, acute kidney injury, p300, garcinol, acetylation

## Abstract

Emerging evidence suggests that epigenetic mechanisms such as histone modification are crucially involved in the pathophysiology of acute kidney injury (AKI). The histone acetyltransferase p300 regulates several biological processes through the acetylation of histones or transcription factors. However, the role of p300 in cisplatin-induced AKI remains poorly understood. Therefore, we investigated the effects of garcinol, a potent p300 inhibitor, on cisplatin-induced AKI and explored the mechanisms. Administration of garcinol significantly reversed the upregulation of p300 and increased acetylation of histone H3, along with amelioration of renal dysfunction and histopathological injury in the kidneys of cisplatin-injected mice. Garcinol also attenuated oxidative stress and reduced expression of pro-oxidant enzymes. In addition, garcinol reduced the elevated production of cytokines and chemokines and suppressed immune cell accumulation together with downregulation of vascular adhesion molecules. These beneficial effects of garcinol were associated with a reduction in acetylation of the p65 subunit of nuclear factor kappa-B. Further, garcinol significantly inhibited apoptosis and caspase-3 activation, with a decrease in p53 acetylation in cisplatin-injected mice. Taken together, we demonstrated that the inhibition of p300 by garcinol ameliorated cisplatin-induced renal injury, presumably through epigenetic mechanisms. These results suggest that garcinol might be a potential preventive agent for cisplatin-induced AKI.

## 1. Introduction

Cisplatin is one of the most potent chemotherapy medications and is widely used for the therapy of several types of cancer [1]. However, its clinical application has been limited due to its severe side effects. Nephrotoxicity is the main therapeutic limitation of cisplatin, affecting about a third of patients undergoing cisplatin therapy [2]. Cisplatin-induced nephrotoxicity is frequently manifested as acute kidney injury (AKI). AKI is defined as an abrupt decline in renal function, which is often associated with structural renal damage. Although several approaches, such as high volume hydration with saline and administration of mannitol, have been used for prevention of the renal complication of cisplatin therapy, their efficacy and safety remain insufficient [2]. Therefore, there is a high unmet medical need for the development of novel therapeutic agents that protect against cisplatin-induced AKI.

The pathophysiology of cisplatin-induced AKI is highly complex [2,3,4]. Although its exact mechanisms remain incompletely understood, accumulating evidence suggests that oxidative stress, inflammatory responses, and tubular cell apoptosis are involved in the pathophysiology of the disease [2,3,4]. Among them, oxidative stress is considered as a critical pathogenic factor for cisplatin-induced AKI. It is known that increased production of reactive oxygen species (ROS) during cisplatin treatment activates multiple signaling cascades, leading to damage and death of renal tubular epithelial cells [5,6]. In addition, a robust inflammatory response triggered by cisplatin contributes to renal functional and structural deterioration. It was reported that genetic or pharmacological suppression of tumor necrosis factor-α (TNF-α) effectively ameliorated cisplatin-induced renal injury [7]. Besides proinflammatory cytokines, chemokines are excessively produced during cisplatin-induced AKI and recruit proinflammatory cells, such as neutrophils and macrophages, into injured tissues [8,9]. Excessive accumulation of proinflammatory cells can further aggravate renal injury. Moreover, apoptosis of renal tubular epithelial cells has attracted much attention of researchers in the study of the mechanism by which cisplatin induces kidney damage [2,3,4]. Various substances have been reported to attenuate renal injury caused by cisplatin by inhibiting apoptosis [10,11,12].

Emerging evidence suggests that epigenetic mechanisms, such as histone modification, are crucially involved in the pathogenesis of AKI [13]. Particularly, histone acetylation is one of the most studied epigenetic modulations in AKI and involves the addition of an acetyl group on the lysine residues of histone proteins [14]. This process induces relaxation of the chromatic structure and thereby promotes recruitment of transcription factors to regulatory regions of genes. Levels of histone acetylation are modulated by histone acetyltransferases (HATs) and histone deacetylases (HDACs). Among members of HATs, p300 is involved in various biological processes, including proliferation, differentiation, and apoptosis, through modulating histone acetylation [15,16]. In addition, p300 can directly bind to transcription factors, such as nuclear factor-κB (NF-κB) and p53, and regulate their activities via acetylation [17,18,19]. Accumulating evidence demonstrates that aberrant expression and/or activity of p300 are critically involved in various pathological conditions, including cancers, neurodegeneration, and inflammatory diseases [20,21,22,23]. It has also been shown that p300 promotes oxidative stress and inflammation in animal models of diabetic nephropathy [24,25,26]. However, the role of p300 in cisplatin-induced AKI remains incompletely understood. Garcinol, a polyisoprenylated benzophenone derivative, is one of the major active constituents isolated from the *Garcinia indica* fruit rind and is known as a potent p300 inhibitor [27,28]. Therefore, in this study, we investigated whether inhibition of p300 by garcinol protects against cisplatin-induced renal injury.

## 2. Materials and Methods

### 2.1. Animal Procedures

Seven-week-old male C57BL/6N mice were obtained from HyoSung Science Inc. (Daegu, Korea) and kept at 20–24 °C and 55% humidity for 1 week. The mice were assigned into 3 groups (*n* = 8 per group): vehicle (Veh), cisplatin (CP), and cisplatin plus garcinol (CP+Gar). The CP group was given a single intraperitoneal injection of cisplatin (20 mg/kg in 0.9% saline; Sigma-Aldrich, St. Louis, MO, USA). An equal volume of the vehicle was injected intraperitoneally into the Veh group. To investigate the effect of garcinol (Abcam, Cambridge, MA, USA) on cisplatin-induced nephrotoxicity, the CP+Gar group was intraperitoneally injected with garcinol (10 mg/kg) for 4 consecutive days, starting from 1 day prior to cisplatin injection. The doses of garcinol and cisplatin were chosen based on the results of previous studies [29,30,31]. All mice were sacrificed 72 h after cisplatin injection. All animal experiments were performed in accordance with the Institutional Animal Care and Use Committee of the Daegu Catholic University Medical Center (approval number: DCIAFCR-200626-13-Y).

### 2.2. Evaluation of Renal Function

Plasma levels of creatinine and blood urea nitrogen (BUN) were analyzed using a creatinine assay kit (BioAssay Systems, Hayward, CA, USA) and a BUN assay kit (Thermo Fisher Scientific, Waltham, MA, USA), respectively, according to the manufacturer’s protocol.

### 2.3. Measurement of Plasma Cytokines

Plasma TNF-α and interleukin-6 (IL-6) levels were measured using standard quantitative sandwich ELISA kits (R&D Systems, Minneapolis, MN, USA) according to the manufacturer’s protocol.

### 2.4. Evaluation of Oxidative Stress

Renal malondialdehyde (MDA) levels were measured using a colorimetric/fluorometric assay kit (Sigma-Aldrich) according to the manufacturer’s protocol. Renal levels of reduced glutathione (GSH) and oxidized glutathione (GSSG) were analyzed using a colorimetric detection kit (Enzo Life Sciences, Farmingdale, NY, USA) according to the manufacturer′s protocol.

### 2.5. Histological Analysis and Immunohistochemical Staining

Kidney tissues were immediately fixed in 10% formalin and then dehydrated in graded series of ethanol. After dehydration, the tissues were cleared in xylene and embedded in paraffin. Thin sections (4 μm) were stained with hematoxylin and eosin (H&E) or periodic acid–Schiff (PAS). The severity of tubular injury was scored semiquantitatively by estimating the percentage of damaged area: 0, 0%; 1, ≤10%; 2, 11–25%; 3, 26–45%; 4, 46–75%; and 5, 76–100% [32,33]. Tubular injury was analyzed in 5 randomly chosen fields at ×400 magnification per kidney sample. For immunohistochemical staining, paraffin-embedded sections were deparaffinized and rehydrated using standard methods. Antigen retrieval was performed using sodium buffer (pH 6.0). The sections were treated with 3% hydrogen peroxidase to block endogenous peroxidase activity. After washing, the sections were incubated with a primary antibody and then probed with a secondary antibody. The primary antibodies used for immunohistochemical staining were as follows: anti-p300 (Santa Cruz Biotechnology, Santa Cruz, CA, USA), anti-neutrophil gelatinase-associated lipocalin (NGAL; Santa Cruz Biotechnology), anti-kidney injury molecule-1 (KIM-1; Abcam), anti-galectin-3 (Abcam), anti-CD4 (Abcam), or anti-4-hydroxynonenal (4-HNE; Abcam) antibodies. Images were visualized and captured using a confocal microscope (Nikon, Tokyo, Japan). The percentage of stained areas was analyzed in 5 arbitrarily chosen fields at ×400 magnification per kidney sample using i-Solution DT software (IMTechnology, Vancouver, BC, Canada). The number of galectin-3 or CD4-stained cells was counted in 5 arbitrarily chosen fields at ×400 magnification per kidney sample.

### 2.6. Immunofluorescent Staining

To detect the brush border of proximal tubule, the kidney sections were stained with fluorescein isothiocyanate (FITC)-labeled lotus tetragonolobus lectin (LTL; Vector Laboratories, Burlingame, CA, USA). To identify neutrophils, the sections were probed with anti-Ly6B.2 antibody (Abcam) and then incubated with a secondary antibody. Nuclei were stained with 4′,6-diamidino-2-phenylindole (DAPI). Images were visualized and captured using a confocal microscope (Nikon). The percentage of stained areas was determined in 5 arbitrarily chosen fields at ×400 magnification per kidney sample. The number of Ly6B.2-stained cells was counted in 5 arbitrarily chosen fields at ×400 magnification per kidney sample.

### 2.7. Immunoblot Analysis

Total proteins were extracted from kidney samples with a lysis buffer (Sigma-Aldrich). A total of 30 μg proteins from each sample were resolved by sodium dodecyl sulfate polyacrylamide gel electrophoresis (SDS-PAGE). Separated proteins were transferred onto a nitrocellulose membrane. The membrane was incubated with a primary antibody and then probed with a horseradish peroxidase-conjugated secondary antibody. The primary antibodies used in the present study were as follows: anti-acetyl-Histone H3 (Lys18; Cell Signaling, Danvers, MA, USA), anti-acetyl-Histone H3 (Lys27; Abcam), anti-acetyl-Histone H3 (Lys9; Cell Signaling), anti-nicotinamide adenine dinucleotide phosphate oxidase 4 (NOX4; Novus Biologicals, Littleton, CO, USA), anti-intercellular adhesion molecule-1 (ICAM-1; Santa Cruz Biotechnology), anti-nuclear factor-κB (NF-κB) p65 (Cell Signaling), anti-acetyl-NF-κB p65 (Lys310; Cell Signaling), anti-cleaved caspase-3 (Cell Signaling), anti-cleaved poly(ADP-ribose) polymerase-1 (cleaved PARP-1; Cell Signaling), anti-p53 (Cell Signaling), anti-acetyl-p53 (Lys382; Cell Signaling), and anti-glyceraldehyde-3-phosphate dehydrogenase (GAPDH; Cell Signaling) antibodies. GAPDH was used as an internal control. The protein bands were visualized using enhanced chemiluminescence (ECL) reagents (Thermo Fisher Scientific) and were analyzed using the iBright™ CL1500 imaging system (Thermo Fisher Scientific).

### 2.8. Real-Time Reverse Transcription Polymerase Chain Reaction (RT-PCR)

Total RNA was isolated from kidney samples using the TRIzol reagent (Thermo Fisher Scientific) and reverse-transcribed into cDNA using the RNA to cDNA EcoDry™ Premix kit (TaKaRa, Tokyo, Japan) according to the manufacturer′s protocol. Quantification of target cDNA levels was carried out using the Power SYBR Green PCR Master Mix (TaKaRa) and the Thermal Cycler Dice Real-Time System III (TaKaRa). Sequences of primer sets used in the present study are shown in Table 1. The internal reference gene was GAPDH.

### 2.9. Terminal Deoxynucleotidyl Transferase-Mediated Deoxyuridine Triphosphate Nick End Labeling (TUNEL) Assay

The kidney sections were deparaffinized, rehydrated, and permeabilized. TUNEL reaction was performed using a TUNEL assay kit (Roche Diagnostics, Indianapolis, IN, USA) according to the manufacturer′s protocol. Nuclei were stained with DAPI. Images were visualized and captured using a confocal microscope (Nikon). The number of cells stained with TUNEL was counted in 5 randomly chosen fields at ×400 magnification per kidney sample.

### 2.10. Statistical Analysis

Data are presented as mean ± standard error of the mean (SEM). One-way analysis of variance (ANOVA) with Bonferroni’s post hoc tests was used for comparison between groups. A *p* value less than 0.05 was considered statistically significant.

## 3. Results

### 3.1. Garcinol Suppressed Renal Expression of p300 and Histone Acetylation and Ameliorated Renal Dysfunction in Cisplatin-Injected Mice

**Figure 1:**
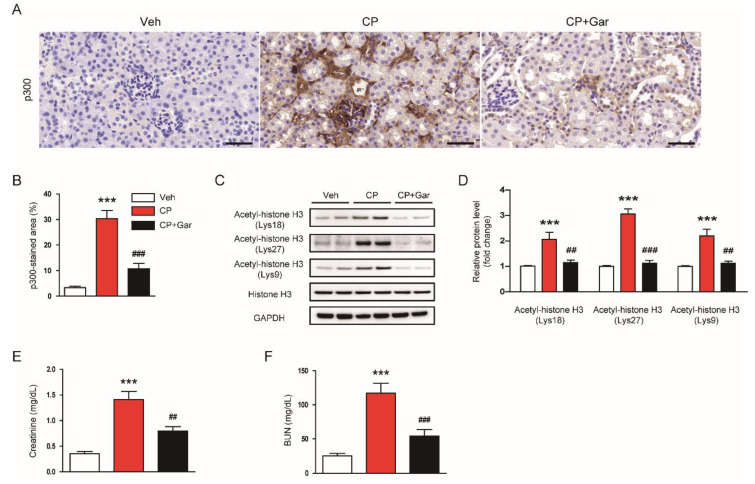
Effect of garcinol on p300 expression, histone acetylation, and renal function in cisplatin-injected mice. Mice were given an intraperitoneal administration with garcinol (10 mg/kg; Gar) daily for four consecutive days, starting from one day prior to cisplatin treatment. All mice were sacrificed 72 h after cisplatin treatment. (**A**) Immunohistochemical staining of kidney tissues for p300. Scale bar = 50 μm. (**B**) Percentage of stained areas for p300. (**C**) Immunoblottings of acetyl-histone H3 (Lys18), acetyl-histone H3 (Lys27), and acetyl-histone H3 (Lys9). (**D**) Quantification of immunoblots for histone H3 acetylation at Lys18, Lys27, and Lys9. Glyceraldehyde-3-phosphate dehydrogenase (GAPDH) was used as a loading control. (**E**) Plasma creatinine levels. (**F**) Blood urea nitrogen (BUN) levels. *n* = 8 per group. *** *p* < 0.001 vs. the vehicle-treated control group (Veh). ^##^ *p* < 0.01 and ^###^ *p* < 0.001 vs. the cisplatin-injected group (CP).

To investigate the role of p300 in cisplatin-induced AKI, we first examined p300 expression in all experimental groups. Immunohistochemical staining showed that cisplatin-injected mice displayed elevated expression of p300 compared to control mice (Figure 1A,B). Consistently, acetylation of lysines 18, 27, and 9 on histone H3, the preferred substrates of p300 [34,35,36,37], was also increased in cisplatin-injected mice (Figure 1C,D), suggesting that cisplatin treatment increases p300 expression and p300-associated histone acetylation. However, administration of garcinol significantly attenuated all these changes (Figure 1A–D). In addition, cisplatin-induced renal impairment, as evidenced by elevated creatinine and BUN levels, was markedly improved by garcinol (Figure 1E,F). Taken together, these results indicate that inhibition of p300 by garcinol ameliorated cisplatin-induced renal dysfunction.

### 3.2. Garcinol Attenuated Histopathological Injury in Cisplatin-Injected Mice

**Figure 2:**
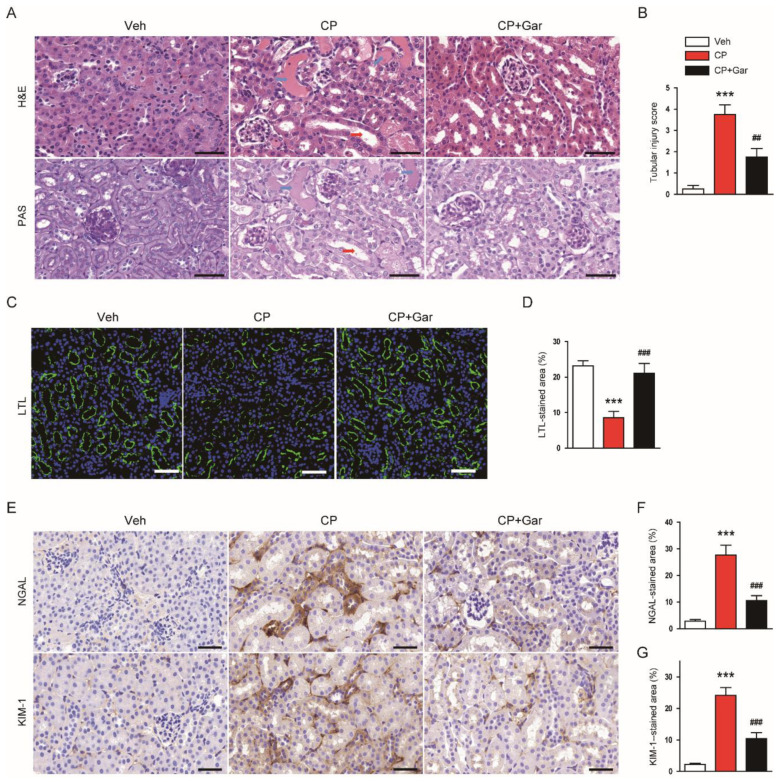
Effect of garcinol on renal structural injury in cisplatin-injected mice. (**A**) Hematoxylin and eosin (H&E) and periodic acid–Schiff (PAS) staining of kidney tissues. Blue arrows indicate tubular cast deposition. Red arrows indicate dilated tubules. Scale bar = 50 μm. (**B**) Tubular injury score. (**C**) Immunofluorescent staining with fluorescein isothiocyanate-conjugated lotus tetragonolobus lectin (LTL) of kidney tissues. Scale bar = 50 μm. (**D**) Percentage of stained areas for LTL. (**E**) Immunohistochemical staining of kidney tissues for neutrophil gelatinase-associated lipocalin (NGAL) or kidney injury molecule-1 (KIM-1). Scale bar = 50 μm. (**F**) Percentage of stained areas for NGAL. (**G**) Percentage of stained areas for KIM-1. *n* = 8 per group. *** *p* < 0.001 vs. Veh. ^##^ *p* < 0.01 and ^###^ *p* < 0.001 vs. CP.

Cisplatin is known to induce renal structural damage, particularly tubular injury, in humans and rodents [1,2,3,4]. Therefore, we next investigated the effect of garcinol on the histological alterations induced by cisplatin treatment. Histological staining showed that cisplatin-injected mice exhibited prominent histopathological alterations, such as cast formation and tubular dilatation, in the kidneys (Figure 2A,B). To visualize the brush border of proximal tubule, the kidney sections were stained with LTL conjugated with FITC. LTL staining revealed a marked loss of brush border in proximal tubules after cisplatin treatment (Figure 2C,D). However, all these alterations were significantly mitigated by garcinol (Figure 2A–D). Further, administration of garcinol also largely suppressed cisplatin-induced induction of NGAL and KIM-1, indicating that garcinol attenuated the cisplatin-induced tubular injury (Figure 2E–G).

### 3.3. Garcinol Suppressed Cisplatin-Induced Oxidative Stress

**Figure 3:**
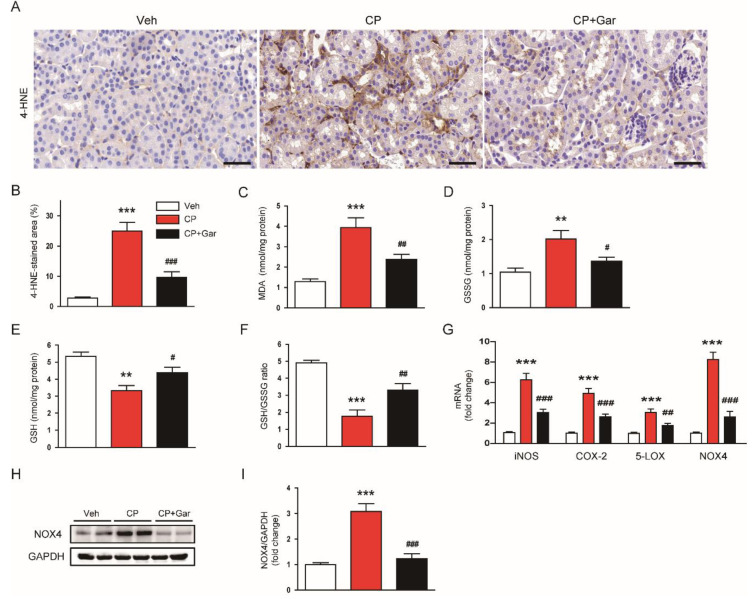
Effect of garcinol on oxidative stress and expression of pro-oxidant enzymes in cisplatin-injected mice. (**A**) Immunohistochemical staining of kidney tissues for 4-hydroxynonenal (4-HNE). Scale bar = 50 μm. (**B**) Percentage of stained areas for 4-HNE. (**C**) Malondialdehyde (MDA) levels. (**D**) Oxidized glutathione (GSSG) levels. (**E**) Reduced glutathione (GSH) levels (**F**) GSH/GSSG ratio. (**G**) The mRNA expression of inducible nitric oxide synthase (iNOS), cyclooxygenase-2 (COX-2), 5-lipoxygenase (5-LOX), and nicotinamide adenine dinucleotide phosphate oxidase 4 (NOX4). (**H**) Immunoblotting of NOX4. (**I**) Quantification of immunoblot for NOX4. *n* = 8 per group. ** *p* < 0.01 and *** *p* < 0.001 vs. Veh. ^#^ *p* < 0.05, ^#^^#^ *p* < 0.01, and ^#^^##^ *p* < 0.001 vs. CP.

Oxidative stress has been recognized as a main contributing factor to cisplatin-induced AKI [2,3,4]. To evaluate whether garcinol suppresses cisplatin-induced oxidative stress, the kidney sections were stained with an antibody against 4-HNE, a well-known by-product of lipid peroxidation [38,39], in all experimental groups. We found that 4-HNE-stained area was markedly increased in cisplatin-injected mice compared to the control group (Figure 3A,B). Cisplatin treatment also increased MDA (Figure 3C) and GSSG (Figure 3D) levels in the kidneys. GSH depletion (Figure 3E) and a reduction of GSH/GSSG ratio (Figure 3F) were also observed in cisplatin-injected mice. However, all these changes were significantly reversed by garcinol (Figure 3A–F).

We next examined the effect of garcinol on expression of pro-oxidant enzymes. Administration of garcinol significantly suppressed mRNA expression of inducible nitric oxide synthase (iNOS), cyclooxygenase-2 (COX-2), 5-lipoxygenase (5-LOX), and NOX4 in the kidneys of cisplatin-injected mice (Figure 3G). Among them, NOX4 is a major source of reactive oxygen species (ROS) production in the kidney and plays a key role in cisplatin-induced oxidative stress [5]. Immunoblot analysis confirmed that elevated protein level of NOX4 after cisplatin treatment was also significantly reduced by garcinol (Figure 3H,I). Collectively, these findings suggest that inhibition of p300 by garcinol suppressed cisplatin-induced oxidative stress, potentially through downregulating expression of pro-oxidant enzymes.

### 3.4. Garcinol Attenuated Cisplatin-Induced Inflammatory Responses

**Figure 4:**
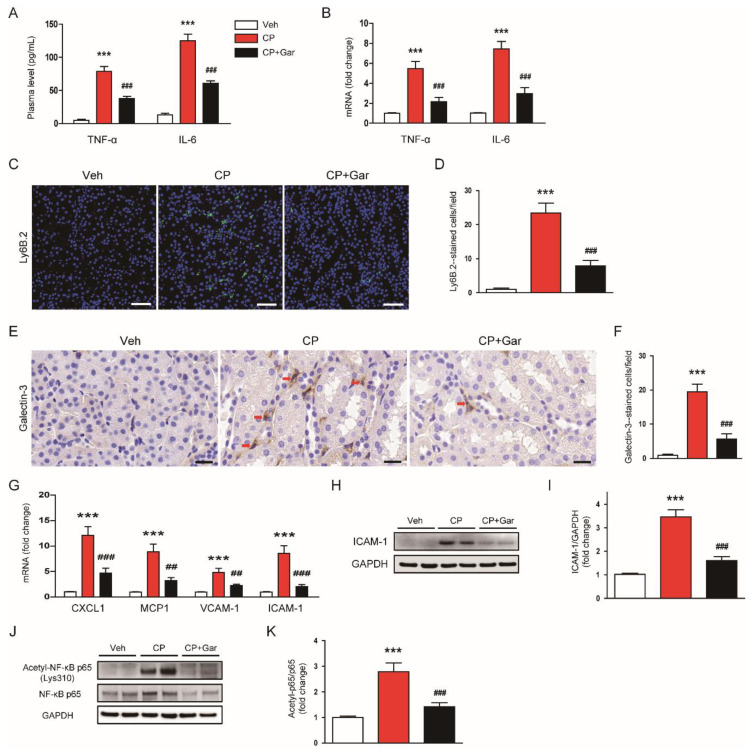
Effect of garcinol on cytokine production, immune cell accumulation, acetylation of nuclear factor-κB (NF-κB) p65 in cisplatin-injected mice. (**A**) Plasma levels of tumor necrosis factor-α (TNF-α) and interleukin-6 (IL-6). (**B**) The mRNA expression of TNF-α and IL-6. (**C**) Immunofluorescent staining of kidney tissues for Ly6B.2. Scale bar = 50 μm. (**D**) Number of Ly6B.2-stained cells. (**E**) Immunohistochemical staining of kidney tissues for galectin-3. Scale bar = 50 μm. (**F**) Number of galectin 3-stained cells. (**G**) The mRNA expression of chemokine (C-X-C motif) ligand 1 (CXCL1), monocyte chemoattractant protein-1 (MCP-1), vascular cell adhesion molecule-1 (VCAM-1), and intercellular adhesion molecule-1 (ICAM-1). (**H**) Immunoblotting of ICAM-1. (**I**) Quantification of immunoblot for ICAM-1. (**J**) Immunoblotting of acetyl-NF-κB p65 (Lys310). (**K**) Quantification of immunoblot for acetyl-NF-κB p65. *n* = 8 per group. *** *p* < 0.001 vs. Veh. ^##^ *p* < 0.01 and ^###^ *p* < 0.001 vs. CP.

Previous studies have reported that cisplatin-injected animals exhibited elevated levels of proinflammatory cytokines and increased accumulation of immune cells in the kidneys [8,9]. As expected, we found that cisplatin treatment increased plasma levels of TNF-α and IL-6, indicating that cisplatin induced systemic inflammation (Figure 4A). Renal mRNA levels of these cytokines were also increased by cisplatin treatment (Figure 4B). However, these increases were significantly suppressed by garcinol (Figure 4A,B). Additionally, garcinol reduced the numbers of Ly6B.2 or galectin-3-stained cells in the kidneys, indicating that immune cell accumulation was suppressed by garcinol (Figure 4C–F). As chemokines and vascular adhesion molecules play key roles in recruitment of immune cells to inflamed tissues [2,3], we next examined the effects of garcinol on their expression levels in the kidneys. We found that garcinol largely attenuated the elevated expression of chemokine (C-X-C motif) ligand 1 (CXCL1), monocyte chemoattractant protein-1 (MCP-1), vascular cell adhesion molecule-1 (VCAM-1), and ICAM-1 (Figure 4G). An increase in protein level of ICAM-1 after cisplatin treatment was also confirmed by immunoblotting (Figure 4H,I). However, administration of garcinol significantly attenuated these changes induced by cisplatin (Figure 4C–I).

NF-κB is an essential transcription factor that modulates oxidative stress and inflammation [2]. In addition, p300 can induce acetylation of lysine 310 of NF-κB p65, which is required for its full transcriptional activity [17,18]. Thus, we next examined the effects of garcinol on acetylation of NF-κB p65 in the kidneys of cisplatin-injected mice. Cisplatin treatment increased acetylation of lysine 310 of NF-κB p65, which was significantly inhibited by garcinol (Figure 4J,K). Altogether, these results suggest that inhibition of p300 by garcinol suppressed cytokine production and immune cell accumulation, presumably through suppressing acetylation of NF-κB p65, in cisplatin-injected mice.

### 3.5. Garcinol Inhibited Cisplatin-Induced Apoptosis

**Figure 5:**
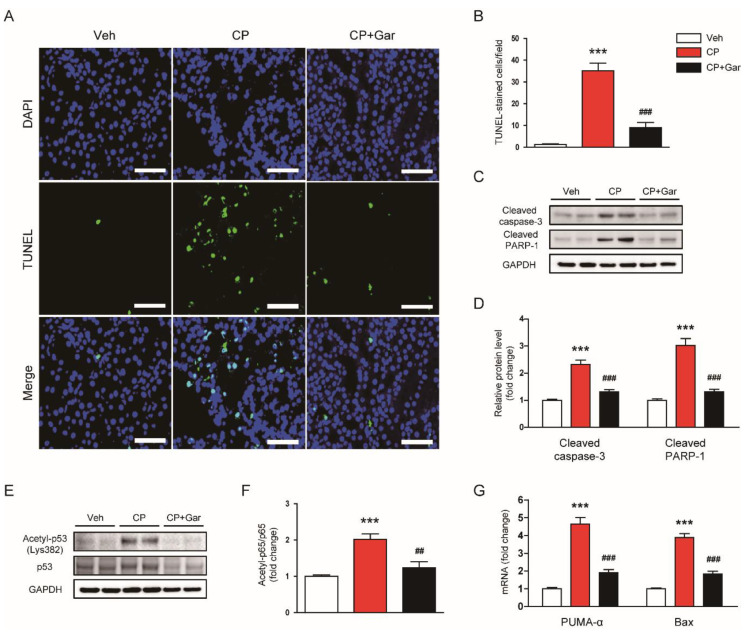
Effect of garcinol on apoptotic cell death in cisplatin-injected mice. (**A**) Terminal deoxynucleotidyl transferase-mediated deoxyuridine triphosphate nick end labeling (TUNEL) staining of kidney tissues. Scale bar = 50 μm. (**B**) Number of cells stained with TUNEL. (**C**) Immunoblottings of cleaved caspase-3 and cleaved poly(ADP-ribose) polymerase-1 (PARP-1). (**D**) Quantification of immunoblots for cleaved caspase-3 and cleaved PARP-1. (**E**) Immunoblottings of acetyla-p53 (Lys382) and p53. (**F**) Quantification of immunoblot for acetyla-p53. (**G**) The mRNA expression of p53-upregulated modulator of apoptosis (PUMA) and Bax. *n* = 8 per group. *** *p* < 0.001 vs. Veh. ^##^ *p* < 0.01 and ^###^ *p* < 0.001 vs. CP.

Tubular cell apoptosis is also a critical contributing factor to cisplatin-induced renal injury [2,3,4]. Thus, we next examined the effect of garcinol on cisplatin-induced apoptosis of tubular epithelial cells. TUNEL assay showed that cisplatin treatment markedly increased the number of apoptotic cells in the kidneys (Figure 5A,B). However, administration of garcinol significantly inhibited cisplatin-induced apoptosis, as evidenced by a reduced number of cells stained with TUNEL (Figure 5A,B). Increased levels of cleaved forms of caspase-3 and PARP-1 were also attenuated by garcinol (Figure 5C,D).

Given that p53 is a key modulator for apoptosis and its acetylation by p300 can regulate its transcriptional activity [19], we next examined the effects of garcinol on p53 acetylation. We observed that acetylation of lysine 382 of p53 was increased along with upregulation of p53-upregulated modulator of apoptosis-α (PUMA-α) and Bax after cisplatin treatment (Figure 5E–G). However, these changes were significantly reversed by garcinol (Figure 5E–G). Collectively, these results suggest that inhibition of p300 by garcinol inhibited apoptotic death of tubular epithelial cells, presumably through suppressing acetylation of p53.

## 4. Discussion

**Figure 6:**
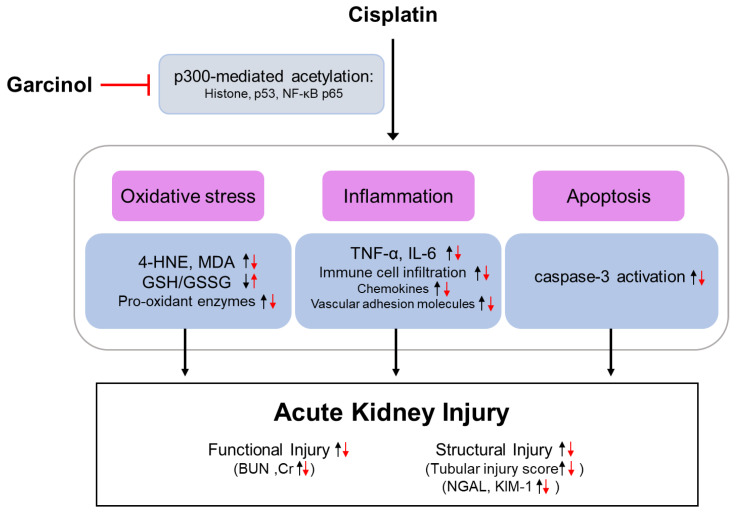
Schematic summary of key findings of the present study. Inhibition of p300 by garcinol attenuated cisplatin-induced functional and structural renal injury through suppressing oxidative stress, inflammation, and apoptosis. These effects were associated with a reduction in acetylation of NF-κB p65 and p53 as well as histone acetylation.

In the present study, we aimed to investigate whether inhibition of p300 by garcinol protected against cisplatin-induced renal injury. We demonstrated that inhibition of p300 by garcinol ameliorated cisplatin-induced functional and histological injury, potentially through suppressing histone acetylation (Figure 6). Mechanistically, oxidative stress, inflammation, and apoptosis induced by cisplatin treatment were attenuated by garcinol. These favorable effects of garcinol were also associated with a reduction in acetylation of NF-κB p65 and p53. Although we cannot exclude the possibility of the involvement of other targets, our findings suggest that the inhibitory effect of garcinol on cisplatin-induced renal injury is mediated, at least in part, by p300 inhibition.

Histone acetylation is characterized by the addition of an acetyl group to the lysine of histones by HATs [14]. Accumulating evidence suggests that various types of AKI, including ischemia–reperfusion injury [40], endotoxin-induced injury [41], or folic acid-induced injury [42], are associated with changes of histone acetylation in response to renal stress. Among members of HATs, p300 has been shown to play an important role in the pathophysiology of various diseases, including cancers, neurodegeneration, and inflammatory diseases [20,21,22,23]. However, the role of p300 in cisplatin-induced renal injury remains unclear. In this study, we observed that cisplatin treatment increased p300 expression along with acetylation of lysines 18, 27, and 9 on histone H3 in the kidneys. Lysines 18 and 27 on histone H3 are the main acetylation targets of p300 [34,35]. Lysine 9 on histone H3 can also be acetylated by p300 [36,37]. However, these changes in p300 level and histone acetylation were significantly attenuated by garcinol. Further, administration of garcinol ameliorated cisplatin-induced functional and histological injury, especially tubular injury, in the kidneys. Acetylation of histones can induce relaxation of the chromatic structure and thereby facilitate the binding of transcription factors to regulatory regions of genes associated with oxidative stress, inflammation, or apoptosis [13,14]. Garcinol has been demonstrated as a potent inhibitor of p300 in vitro and in vivo [27,28]. Therefore, our findings suggest that inhibition of p300 by garcinol protects against cisplatin-induced renal injury, presumably through modulating histone acetylation. 

Excessive oxidative stress has been recognized as one of the hallmarks of cisplatin-induced AKI [2,3,4]. In our study, we found that increased oxidative stress after cisplatin treatment was significantly suppressed by garcinol, as reflected by a reduction in 4-HNE, MDA, and GSSG levels and an increase in GSH level and GSH/GSSG ratio. Administration of garcinol attenuated upregulation of iNOS, COX-2, 5-LOX, and NOX4 in the kidneys of cisplatin-injected mice. Consistently, previous studies have shown the inhibitory effects of garcinol on ROS generation, NO production, and resultant oxidation of lipid and protein [43,44]. NO produced by iNOS has been implicated in cisplatin-induced oxidative injury to kidney tissues [45]. Modulation of arachidonic acid metabolism by COX-2 and 5-LOX also contributes to renal oxidative stress induced by cisplatin [46,47]. Additionally, it was reported that garcinol inhibited expression of iNOS and COX-2 in lipopolysaccharide-stimulated microglia [48] and IL-1β-treated chondrocytes [30]. 5-LOX pathway was also suppressed by garcinol [49]. Emerging evidence suggests that besides these pro-oxidant enzymes, NOX4 is the main source of ROS generation in the kidney and plays a crucial role in cisplatin-induced oxidative stress [5]. Further, p300 has been shown to promote oxidative stress and tissue injury in animal models of diabetic kidney disease [25,26]. Therefore, these results suggest that inhibition of p300 by garcinol contributes to suppression of cisplatin-induced oxidative stress, presumably through downregulating pro-oxidant enzymes.

Inflammation also contributes to the pathophysiology of cisplatin-induced renal injury [2,3,4]. In cisplatin-induced AKI, infiltrated immune cells and tubular epithelial cells secrete marked amounts of cytokines and chemokines, leading to aggravation of tissue injury. In this study, we observed that an increase in circulating levels of TNF-α and IL-6 after cisplatin treatment was significantly reduced by garcinol. Administration of garcinol also reduced renal mRNA levels of both cytokines. These results indicate the inhibitory effects of garcinol on systemic and local inflammation. Earlier studies have recognized that TNF-α is a key mediator in cisplatin-induced inflammatory responses [2,3,4]. Genetic or pharmacological suppression of TNF-α effectively ameliorated inflammation and tissue injury in cisplatin-injected mice [7]. IL-6 also has been shown to play an important role in cisplatin-induced inflammation [50]. Consistent with our findings, previous studies have shown that garcinol suppressed cytokine production in lipopolysaccharide-stimulated microglia [48], IL-1β-treated chondrocytes [30], and kidneys from mice subjected to unilateral ureteral obstruction [51]. In addition, we found that administration of garcinol suppressed accumulation of neutrophils and macrophages along with downregulation of chemokines and vascular adhesion molecules. Immune cell infiltration is commonly observed in cisplatin-induced AKI, contributing to additional tissue injury [8,9]. A variety of chemokines, such as CXCL1, MCP-1, and MIF, contribute to the recruitment of immune cells into the tissues [2]. Vascular adhesion molecules, such as VCAM-1 and ICAM-1, are predominantly expressed in endothelial cells and also facilitate extravasation of immune cells into the tissues [3]. Given that p300 is importantly involved in the development of renal inflammation in diabetic kidney disease [24,25,26], our findings suggest that inhibition of p300 by garcinol is responsible for the suppression of cisplatin-induced inflammatory responses, presumably through inhibiting cytokine production and immune cell accumulation.

NF-κB is a key transcription factor that regulates oxidative stress and inflammation [2]. Expression of numerous cytokines, chemokines, and vascular adhesion molecules is modulated by NF-κB pathway. It has been demonstrated that p300 can induce acetylation of lysine 310 of NF-κB p65, which is required for its full transcriptional activity [17,18]. Previously, we and others have reported that cisplatin treatment led to an increase in acetylation of NF-κB p65 at lysine 310 in mouse kidney and tubular epithelial cells [52,53]. Here, we observed that acetylation of NF-κB p65 at lysine 310 was increased in cisplatin-injected mice compared to control mice. Interestingly, administration of garcinol reduced the acetylation of NF-κB p65. Consistently, a recent study showed that a reduction in p300-mediated acetylation of p65 was associated with suppression of NF-κB activation, resulting in amelioration of cisplatin-induced AKI [54]. Garcinol was also reported to reduce acetylation of lysine 310 of NF-κB p65 in acute toxic liver injury [55]. It is known that sirtuin 1 (Sirt1) is a member of class III group of HDACs that binds to the p65 subunit of NF-κB and suppresses transcription by deacetylating p65 at lysine 310 [56]. Recently, we showed that pharmacological activation of Sirt1 attenuated cisplatin-induced inflammation through deacetylating NF-κB p65 at lysine 310 [52]. In addition, overexpression of Sirt1 suppressed acetylation of NF-κB p65 at lysine 310 and cytotoxicity in tubular epithelial cells treated with cisplatin [53].

Tubular cell apoptosis is also critically involved in the pathophysiology of cisplatin-induced renal injury [2,3,4]. As a transcription factor, p53 plays a central role in tubular cell apoptosis in cisplatin-induced AKI [4] and can be acetylated and regulated by p300 [19]. Here, we observed that cisplatin treatment increased acetylation of lysine 382 of p53 along with upregulation of its transcriptional target, PUMA-α and Bax, in the kidneys. As expected, apoptosis of tubular epithelial cells, as assessed by TUNEL staining and immunoblotting of cleaved caspase-3 and cleaved PARP-1, was markedly increased after cisplatin treatment. Interestingly, however, administration of garcinol effectively suppressed cisplatin-induced apoptosis, which was accompanied by a reduction in acetylation of p53 at lysine 382. Consistently, previous studies have reported that garcinol exerted anti-apoptotic effects in animal models of various diseases, including obstructive nephropathy [51], acute toxic liver injury [55], myocardial infarction [57], and epilepsy [58]. Besides acetylated p65 subunit of NF-κB, acetylated p53 can also be deacetylated by Sirt1 [59]. Indeed, it has been shown that Sirt1 activation attenuated cisplatin-induced apoptosis and renal injury through deacetylation of p53 [52,60]. Overexpression of Sirt1 in the renal tubules also ameliorated cisplatin-induced apoptosis of tubular epithelial cells [61]. Collectively, these results suggest that inhibition of p300 by garcinol attenuate cisplatin-induced apoptosis, presumably through decreasing acetylation of p53.

## 5. Conclusions

In conclusion, our study demonstrated that inhibition of p300 by garcinol ameliorated cisplatin-induced functional and histological injury through inhibiting oxidative stress, inflammation, and apoptosis, highlighting the importance of p300 in cisplatin-induced AKI. Importantly, these effects were associated with a reduction in acetylation of NF-κB p65 and p53 as well as histone acetylation. These findings support the idea that epigenetic modulation may be a useful therapeutic strategy for AKI. As garcinol is known to sensitize cancer cells to chemotherapeutic agents [62,63], this natural compound might be a useful therapeutic option for both cancer and AKI in cancer patients undergoing cisplatin treatment.

## Figures and Tables

**Table 1:** Primers used for real-time RT-PCR in this study.

Gene	Primer Sequence (5′→3′)	Accession No.
iNOS ^1^	Forward: CGAAACGCTTCACTTCCAA Reverse: TGAGCCTATATTGCTGTGGCT	NM_010927
COX-2 ^2^	Forward: AACCGCATTGCCTCTGAAT Reverse: CATGTTCCAGGAGGATGGAG	NM_011198
5-LOX ^3^	Forward: ATTGTTCCCATTGCCATCCAGCTCA Reverse: TCGTTCTCATAGTAGATGCTCACCA	NM_009662
NOX4 ^4^	Forward: GAACCCAAGTTCCAAGCTCATT Reverse: GGCACAAAGGTCCAGAAATCC	NM_015760
CXCL1 ^5^	Forward: ACCCGCTCGCTTCTCTGT Reverse: CACCTTTTAGCATCTTTTGG	NM_008176
MCP-1 ^6^	Forward: TAAAAACCTGGATCGGAACCAA Reverse: GCATTAGCTTCAGATTTACGGGT	NM_011333
VCAM-1 ^7^	Forward: CCCAGGTGGAGGTCTACTCA Reverse: CAGGATTTTGGGAGCTGGTA	NM_011693
ICAM-1 ^8^	Forward: TTCACACTGAATGCCAGCTC Reverse: GTCTGCTGAGACCCCTCTTG	NM_010493
PUMA-α ^9^	Forward: AGCAGCACTTAGAGTCGCC Reverse: CCTGGGTAAGGGGAGGAGT	NM_133234
Bax	Forward: TGCTACAGGGTTTCATCCAG Reverse: ATCCACATCAGCAATCATCC	NM_007527
GAPDH ^10^	Forward: ACTCCACTCACGGCAAATTC Reverse: TCTCCATGGTGGTGAAGACA	NM_001289726

^1^ Inducuble nitric oxide synthase; ^2^ cyclooxygenase-2; ^3^ 5-lipoxygenase; ^4^ nicotinamide adenine dinucleotide phosphate oxidase 4; ^5^ chemokine (C-X-C motif) ligand 1; ^6^ monocyte chemoattractant protein-1; ^7^ vascular cell adhesion molecule-1; ^8^ intercellular adhesion molecule-1; ^9^ p53-upregulated modulator of apoptosis-α; ^10^ glyceraldehyde-3-phosphate dehydrogenase.
